# A surgical activity model of laparoscopic cholecystectomy for co-operation with collaborative robots

**DOI:** 10.1007/s00464-024-10958-w

**Published:** 2024-06-13

**Authors:** R. Younis, A. Yamlahi, S. Bodenstedt, PM. Scheikl, A. Kisilenko, M. Daum, A. Schulze, P. A. Wise, F. Nickel, F. Mathis-Ullrich, L. Maier-Hein, BP. Müller-Stich, S. Speidel, M. Distler, J. Weitz, M. Wagner

**Affiliations:** 1 Department for General, Visceral and Transplant Surgery, Heidelberg University Hospital, Heidelberg, Germany; 2 National Center for Tumor Diseases (NCT), Heidelberg, Germany; 3 Division of Intelligent Medical Systems (IMSY), German Cancer Research Center (DKFZ), Heidelberg, Germany; 4 Department for Translational Surgical Oncology, National Center for Tumor Diseases, Partner Site Dresden, Dresden, Germany; 5 Centre for the Tactile Internet with Human-in-the-Loop (CeTI), TUD Dresden University of Technology, Dresden, Germany; 6 Surgical Planning and Robotic Cognition (SPARC), Department Artificial Intelligence in Biomedical Engineering (AIBE), Friedrich-Alexander-University Erlangen-Nürnberg, Erlangen, Germany; 7 Department of Visceral, Thoracic and Vascular Surgery, Faculty of Medicine and University Hospital Carl Gustav Carus, TUD Dresden University of Technology, Fetscherstraße 74, 01307 Dresden, Germany; 8 Department of General, Visceral and Thoracic Surgery, University Medical Center Hamburg- Eppendorf, Hamburg, Germany; 9 Department for Abdominal Surgery, University Center for Gastrointestinal and Liver Diseases, Basel, Switzerland

**Keywords:** Collaborative surgical robots, Robot autonomy, Surgical process modeling, Surgical data science, Action recognition, Machine learning

## Abstract

**Background:**

Laparoscopic cholecystectomy is a very frequent surgical procedure. However, in an ageing society, less surgical staff will need to perform surgery on patients. Collaborative surgical robots (cobots) could address surgical staff shortages and workload. To achieve context-awareness for surgeon-robot collaboration, the intraoperative action workflow recognition is a key challenge.

**Methods:**

A surgical process model was developed for intraoperative surgical activities including actor, instrument, action and target in laparoscopic cholecystectomy (excluding camera guidance). These activities, as well as instrument presence and surgical phases were annotated in videos of laparoscopic cholecystectomy performed on human patients (*n* = 10) and on explanted porcine livers (*n* = 10). The machine learning algorithm Distilled-Swin was trained on our own annotated dataset and the CholecT45 dataset. The validation of the model was conducted using a fivefold cross-validation approach.

**Results:**

In total, 22,351 activities were annotated with a cumulative duration of 24.9 h of video segments. The machine learning algorithm trained and validated on our own dataset scored a mean average precision (mAP) of 25.7% and a top *K* = 5 accuracy of 85.3%. With training and validation on our dataset and CholecT45, the algorithm scored a mAP of 37.9%.

**Conclusions:**

An activity model was developed and applied for the fine-granular annotation of laparoscopic cholecystectomies in two surgical settings. A machine recognition algorithm trained on our own annotated dataset and CholecT45 achieved a higher performance than training only on CholecT45 and can recognize frequently occurring activities well, but not infrequent activities.

The analysis of an annotated dataset allowed for the quantification of the potential of collaborative surgical robots to address the workload of surgical staff. If collaborative surgical robots could grasp and hold tissue, up to 83.5% of the assistant’s tissue interacting tasks (i.e. excluding camera guidance) could be performed by robots.

**Supplementary Information:**

The online version contains supplementary material available at 10.1007/s00464-024-10958-w.

## Objectives

The aim of this study was to develop and validate a fine-granular workflow model of surgical activities in laparoscopic cholecystectomy for cooperation with collaborative surgical robots.

## Background

Laparoscopic cholecystectomy (LC) is the gold standard for gallbladder removal, with over 700,000 surgeries performed each year in the United States and over 170,000 in Germany [1, 2]. However, surgical staff shortages pose a serious challenge in our ageing society as less surgical staff will need to perform LC patients [3–5].

As a possible solution, computer vision for LC shows potential to alleviate the workload of the surgical staff with the advent of intraoperative decision support, automated quality control and cooperation with collaborative robots [6–9].

Specifically, in contrast to commercially available teleoperated surgical robots, collaborative surgical robots work alongside the surgeon during conventional laparoscopic surgery by automatically performing surgical tasks, such as camera guidance or suturing bowel anastomosis [10–13]. This hybrid approach has shown promising results in non-medical fields such as industrial assembly lines [14–18].

A key challenge for surgical computer vision is the application of fine-granular surgical workflow recognition in order for collaborative surgical robots to become context-aware [8, 19–22]. To train machine learning (ML) algorithms with surgical knowledge for fine-granular surgical workflow recognition, surgical workflow information needs to be captured and structured in large quantity but data which includes fine-granular surgical workflow annotation for LC is lacking [23–25]. One established dataset, CholecT45 (previously CholecT40, based on videos from the single-center Cholec80 benchmark dataset), uses the concept of action triplets (instrument, verb, target) for surgical action data annotation and was used as a benchmark dataset for surgical workflow recognition in the MICCAI EndoVis challenges CholecTriplet2021 and CholecTriplet2022 [26–30]. However, clear action, verb and target class annotation rules are not publicly available and the used annotation software is proprietary, thus the dataset is neither reproducible nor easily extendable [31, 32]. Also, actor information is not included, meaning that action triplet recognition for each of the surgeon’s and surgical assistant’s hands for the development of individual collaborative robot arms is not possible.

Neumuth et al. proposed to formalize the fine-granular surgical workflow using the surgical activity, which is a quadruplet (actor, instrument, action, target) [33]. This concept was previously introduced for surgical action recognition in LC in the MICCAI EndoVis 2019 HeiChole benchmark challenge [34]. However, this challenge focused on surgical phases and the data used for the action recognition task did not contain information on the actor, instrument or target of an action.

Furthermore, a key step in the development of collaborative autonomous surgical robots is in making the safe preclinical translation of prototypes possible [35, 36]. Despite this, research on surgical action recognition in multiple surgical settings like phantom, ex vivo porcine, in vivo porcine and human cadaver is scarce [37].

To overcome these limitations with regards to activity recognition for collaborative surgical robotics, we aimed to answer the following research questions:

- How can the concept of surgical activity be applied to model laparoscopic cholecystectomy in the surgical settings “in vivo human” and “ex vivo porcine”?
- How can the model be applied to real-world procedures by means of human intelligence, i.e. manual video annotation?
- Can a machine learning algorithm automate activity recognition?

The contributions of this work are threefold: we develop and share a surgical process model of the activity workflow for LC (activity model), annotate and analyze a novel activity dataset on publicly available human LC videos and new ex vivo porcine LC videos with a free and open-source annotation software, and evaluate the performance of a ML algorithm trained on our novel dataset and on CholecT45.

## Materials & methods

### Activity model

#### Model development

The previously introduced surgical activity definition was used for the development of a novel surgical process model for video annotation of LC [33]. Discussion rounds were performed between a board-certified general surgeon, an engineer and two medical students with experience in surgical data annotation until an agreement for the model was found. The following characteristics were defined using practical guidelines defined in previous research:

- Activity definition: modeling actor, instrument, action, and target information in a bottom-up manner as activities with the following definitions [38, 39].
  - Actor: specifies which stakeholder’s hand performs the action.
  - Instrument: specifies with which tool the action is performed by the actor.
  - Action: specifies what is being done by the actor.
  - Target: specifies what tissue is being manipulated.
- Standardization: defining a hierarchical task granularity structure that corresponds to a recent consensus and applying it on videos from start to end including idle time [40].
- Causality: modeling for causal consumption of data, meaning that the future should not be known when observing a given moment during a procedure [39].
- Generalizability: modeling for application to a generic LC procedure to capture procedure variations for the preclinical development of collaborative surgical robot, both on explanted porcine livers and on human patients [38, 40, 41].

#### Class selection

The activity model was populated with classes for each activity feature, for example “right hand of surgeon” for actors, “overholt” for instruments, “blunt dissect” for actions or “Calot triangle” for targets (Supplementary Material, Sect. 1). The selection of classes was based on previous research and the clinical experience of a board certified general surgeon [34, 42]. In total, 7 surgical phases, 3 actors, 24 instruments, 12 actions, 25 targets and binary instrument visibility were selected as possible classes.

### Model validation with manual annotation

#### Data collection

Videos of LC performed on human patients by surgeons in the Hospital Salem (Heidelberg, Germany) present in the publicly available HeiChole benchmark dataset were selected for annotation, amounting to ten videos [34]. This represented videos from a single center in the HeiChole benchmark dataset in order to manage annotation effort and ensure consistency of the data, because only LC with the same trocar placement and using the most popular dissection technique in Germany, the electric hook, were included [43, 44].

Ten videos of LC performed on explanted porcine livers by medical students and surgical residents in the surgical training center of Heidelberg University Hospital (Heidelberg, Germany) were collected for annotation. To manage annotation effort, only LC videos on explanted porcine livers with a length of less than one hour were included [44]. Video segments in which the endoscope was outside of the laparoscopy box were censored with a white frame.

As the HeiChole benchmark dataset is publicly available and all videos were fully anonymized before use, no ethics approval or written consent were needed.

#### Data annotation

The video annotation rules were based on the surgical activity model (Supplementary Material, Sect. 2). The free and open-source ANVIL annotation software was used for annotation [45].

**Fig. 1:**
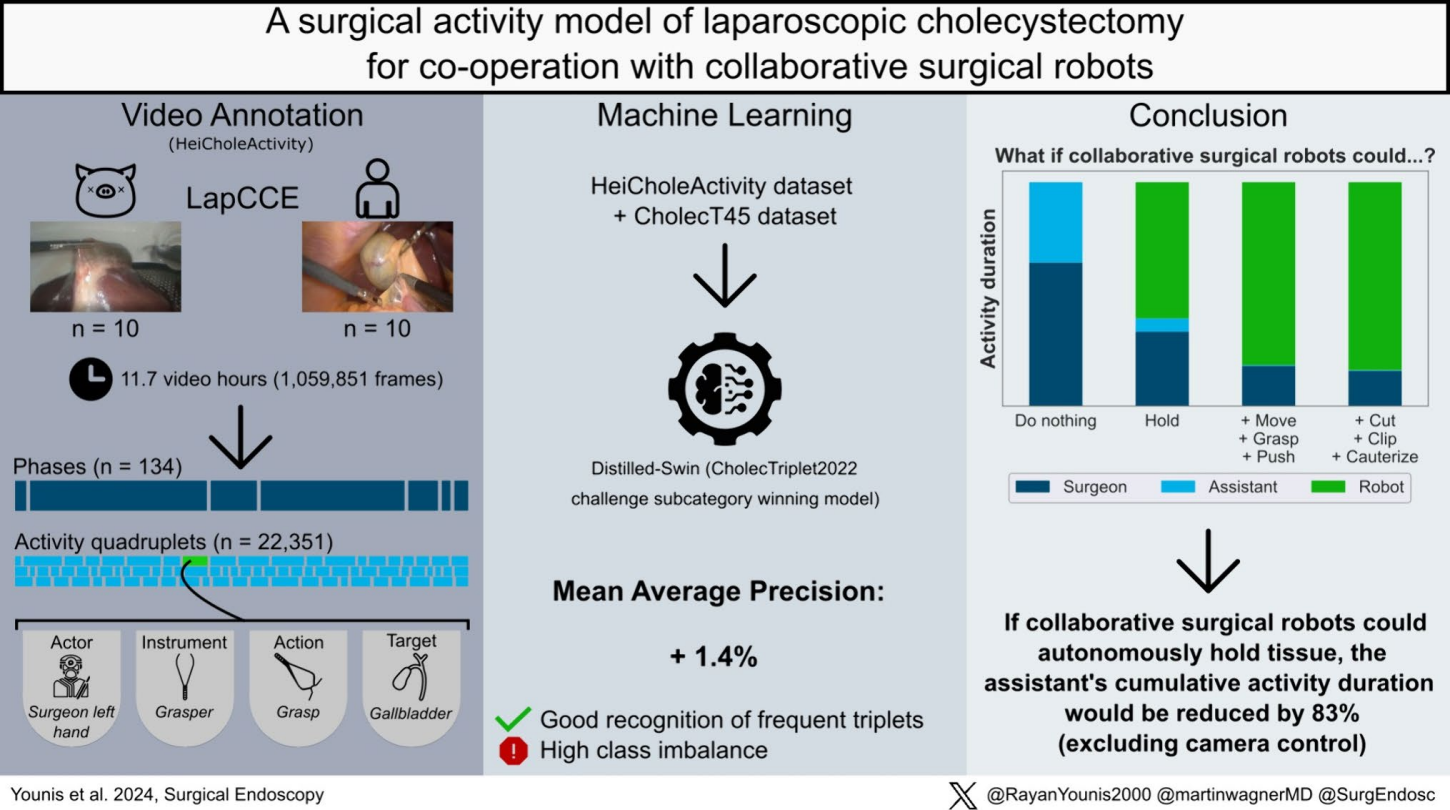
Visual abstract of the study. The left bottom shows an example of an activity: “The surgeon’s left hand used a grasper to grasp the gallbladder from 14:47,50 to 14:49,00 min.”

All videos were converted to 25 frames per second and annotated by one medical student with experience in surgical data annotation and clinical experience as a first assistant in LC (Fig. 1). Class and rule ambiguities that were discovered during the annotation process were resolved by discussions with a board-certified general surgeon. Accordingly, video annotations were systematically updated 11 times in total as the annotation rules were being refined.

### Automatic activity recognition with machine learning

#### Data processing

A machine learning (ML) algorithm was trained and tested for automatic surgical action recognition on the annotated data. A ML algorithm called Distilled-Swin, which ranked first in the action triplet recognition category of the MICCAI EndoVis challenge CholecTriplet2022, the variant (SwinT + MultiD + SelfD) model, was used [29, 46]. Distilled-Swin is a swin transformer model trained through self-distillation, this approach is specifically designed to address challenges related to high number of classes and class imbalance [47].

To use the dataset as input data for ML, it was preprocessed in the following order: invisible activities were removed to limit the introduction of potential class ambiguities. The actor was removed from each activity quadruplet to form action triplets to comply with the algorithm design. All targets of activities with the action “move” were converted to “no target” to reduce potential temporal ambiguities. The individual classes were grouped by clinical similarity, relevance and categorizations in previous literature [26, 30, 34, 48] to reduce the number of occurring triplet combinations to 100 or less, as favored by the algorithm design (Supplementary Material, Sect. 3). Independently, the individual classes of the surgical activity model were also grouped to symbolically fit into the classes used in CholecT45. The dataset’s video frames were sampled at a constant framerate of 3.125 FPS to limit the overall model input to less than 150.000 frames because of hardware limitations, similar to previous works in literature [49].

#### Experiments

Three experiments were carried out with the Distilled-Swin algorithm. First, training and cross-validation were performed on our own dataset using the groupKfold algorithm to avoid data leakage between video sequences [50]. Each video appeared exactly once in the test set across all folds (Table 1). The data were stratified based on the triplet combination to balance the number of classes per fold. The action triplet recognition performance was measured with the mean average precision (mAP) and top *K* = 5 Accuracy (Top-5-Acc) percentages using the ivtmetrics library [49]. Performance differences with different data input were investigated (Table 1).

**Table 1:** Data split for cross-validation using all data

	Fold 0	Fold 1	Fold 2	Fold 3	Fold 4
0	HeiChole17	002exvivo	005exvivo	004exvivo	001exvivo
1	HeiChole18	013exvivo	009exvivo	010exvivo	011exvivo
2	HeiChole20	HeiChole16	HeiChole19	HeiChole14	012exvivo
3	–	HeiChole21	HeiChole22	HeiChole15	014exvivo
4	–	–	–	–	HeiChole13

Second, the algorithm was trained on the combination of our HeiCholeActivity dataset and the CholecT45 dataset and then validated on the official cross-validation split of the CholecT45 dataset [49]. The calculation of the action triplet recognition performance was identical to the first experiment and mAP was measured.

Third, our dataset was separated by actor into three subsets, meaning that each subset included instrument, action and target annotations specific to the surgeon’s left hand, surgeon’s right hand or assistant’s right hand. A Distilled-Swin algorithm was trained on all subsets. The cross-validation of each algorithm was identical to the first experiment and mAP was measured.

## Results

### Activity dataset

**Fig. 2:**
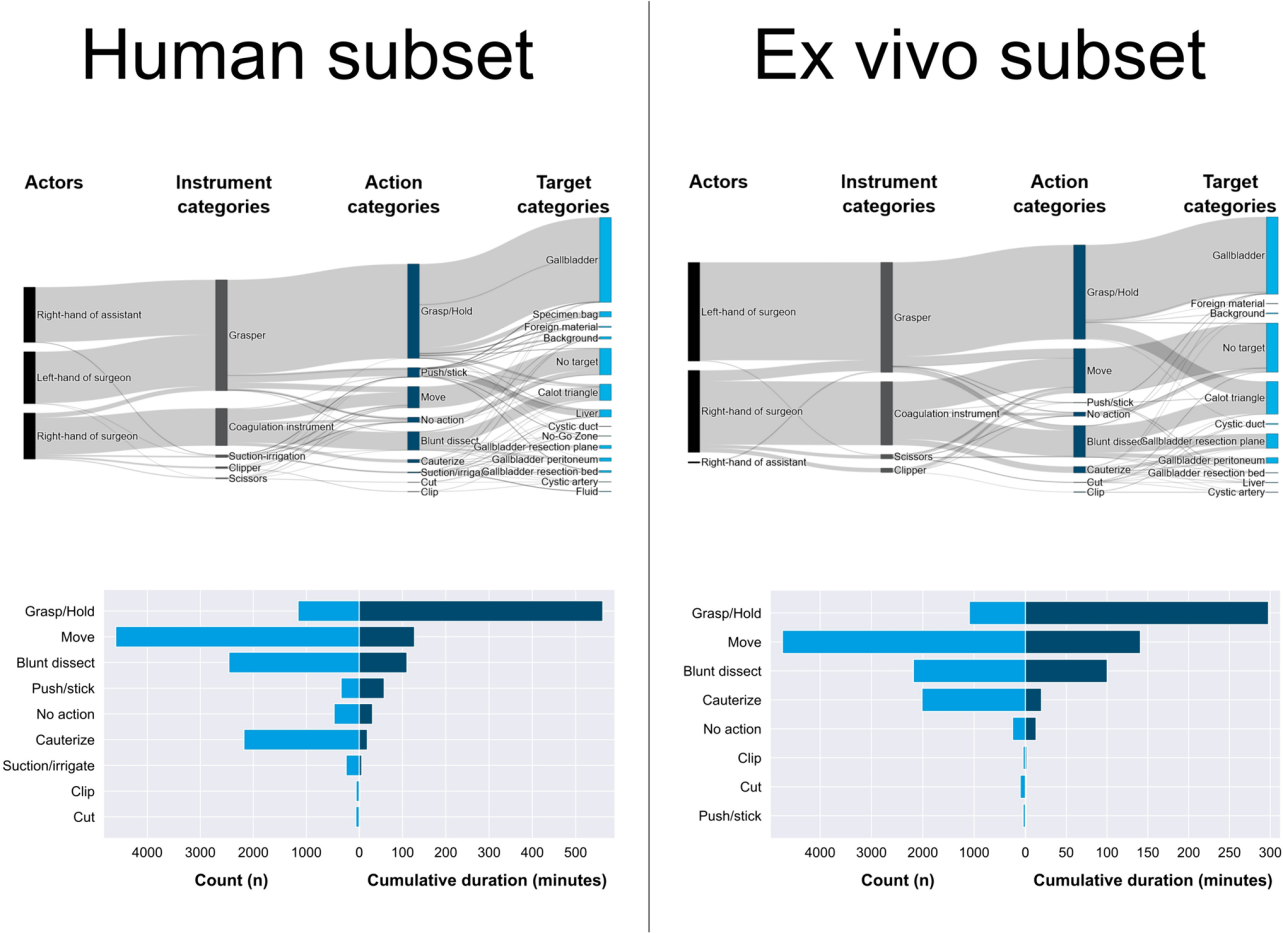
Selection of descriptive statistics of the activity model-based annotated HeiCholeActivity dataset for the human subset (left) and ex vivo porcine subset (right). Top: Cumulative duration of combinations of annotated features within all activities. Bottom: Total amount of activity time segments (light blue) and cumulative duration of activity time segments (dark blue), grouped by action category

Our dataset consists of *n* = 20 videos at 25 FPS with a cumulative duration of 11.7 h or 1,059,851 frames (Fig. 1). The dataset contains 22,351 annotated individual activities, i.e. time segments corresponding to activities, with a cumulative duration of 24.9 h. For the human subset, there were more different targets compared to the ex vivo porcine subset and the addition of the assistant’s hand to the annotation resulted in the actions “grasp” and “hold” having a higher proportion of the overall duration. The cumulative number and cumulative duration of activities for human and ex vivo porcine subsets were differentiated (Fig. 2). Instrument presence was annotated 9,841 times and phases 134 times in total. A proportion of 9.02% of frames contains no visible activity at all. 83.5% of the assistant’s cumulative activity duration consisted of “grasp” and “hold”.

### Automatic action triplet recognition

Our own class reduction method for model input resulted in 5 instrument categories, 9 action categories and 14 target categories which formed 88 different triplet combinations occurring in the dataset. The dataset had a size of 132,482 frames after preprocessing.

In the first experiment with training on our own HeiCholeActivity dataset only with our own class reduction method, the action triplet recognition algorithm scored 25.7% mAP and 85.5% Top-5-Acc using all videos for training and cross-validation. Detailed results of the algorithm cross-validation performance can be seen in Table 2.

**Table 2:**
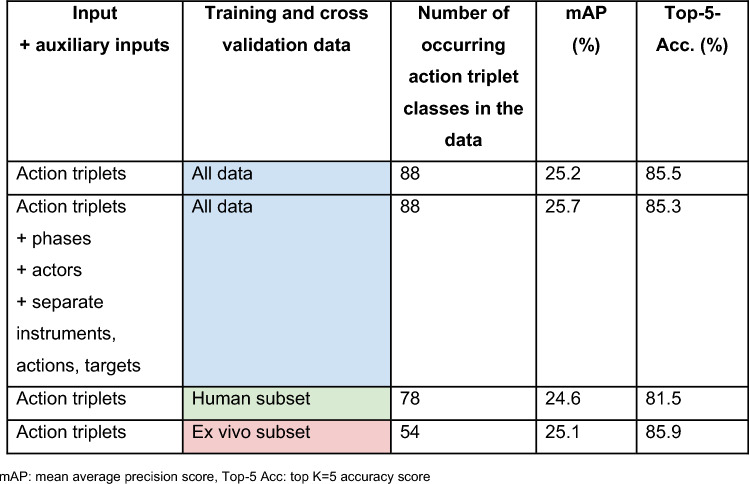
Action triplet recognition algorithm performance using different training data input from the HeiCholeActivity dataset

*mAP* mean average precision score, *Top−5 Acc* top K=5 accuracy score

**Fig. 3:**
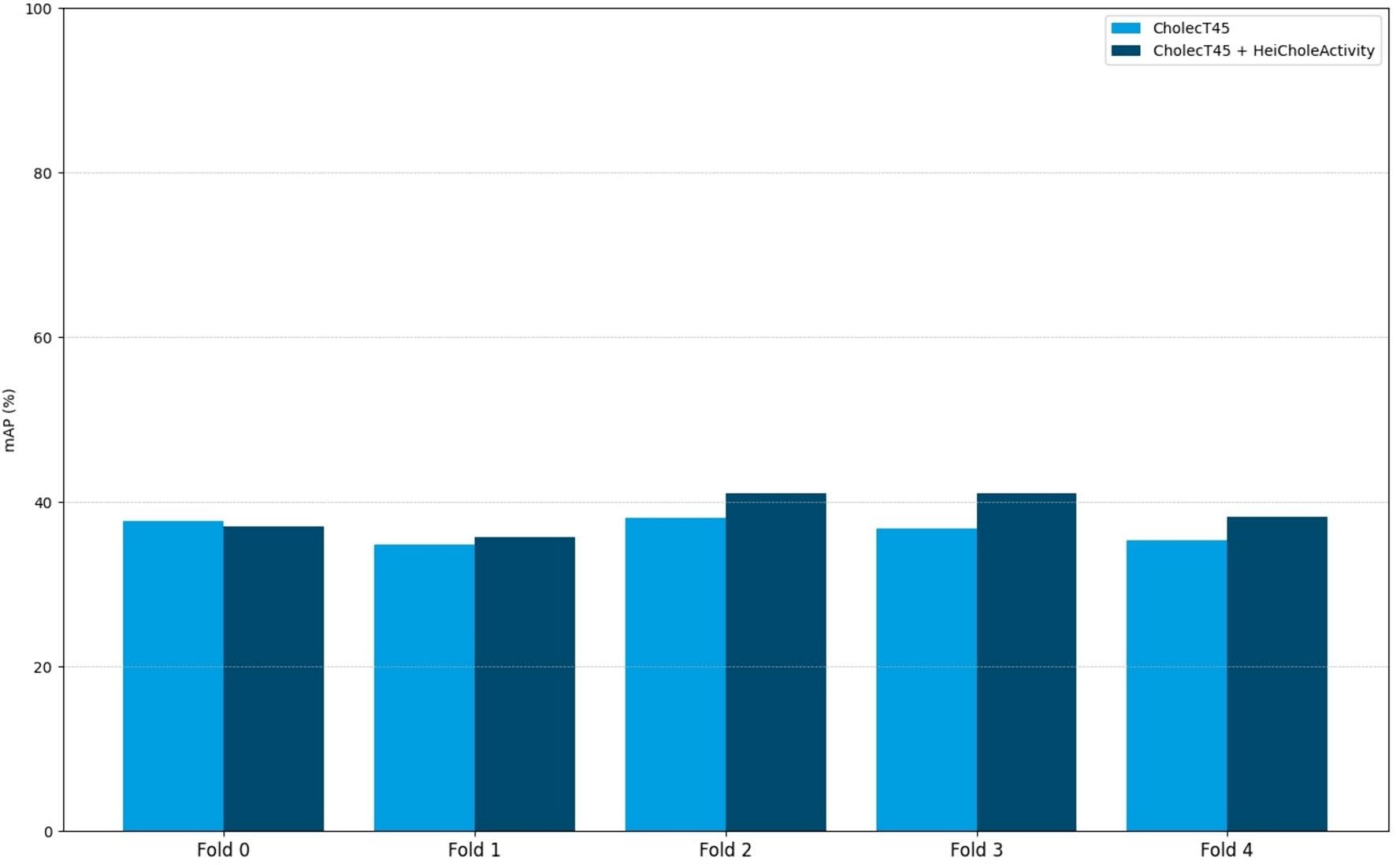
Comparison between the Distilled-Swin algorithm cross-validation performances on CholecT45 using only CholecT45 for training or the combination of CholecT45 and HeiCholeActivity

In the second experiment with training on our own HeiCholeActivity dataset and CholecT45 with CholecT45 triplet classes, the action triplet recognition algorithm scored 37.9% mAP. Figure 3 presents an overview of the per-fold performance compared to algorithm performance using only CholecT45 for training [31, 46].

**Fig. 4:**
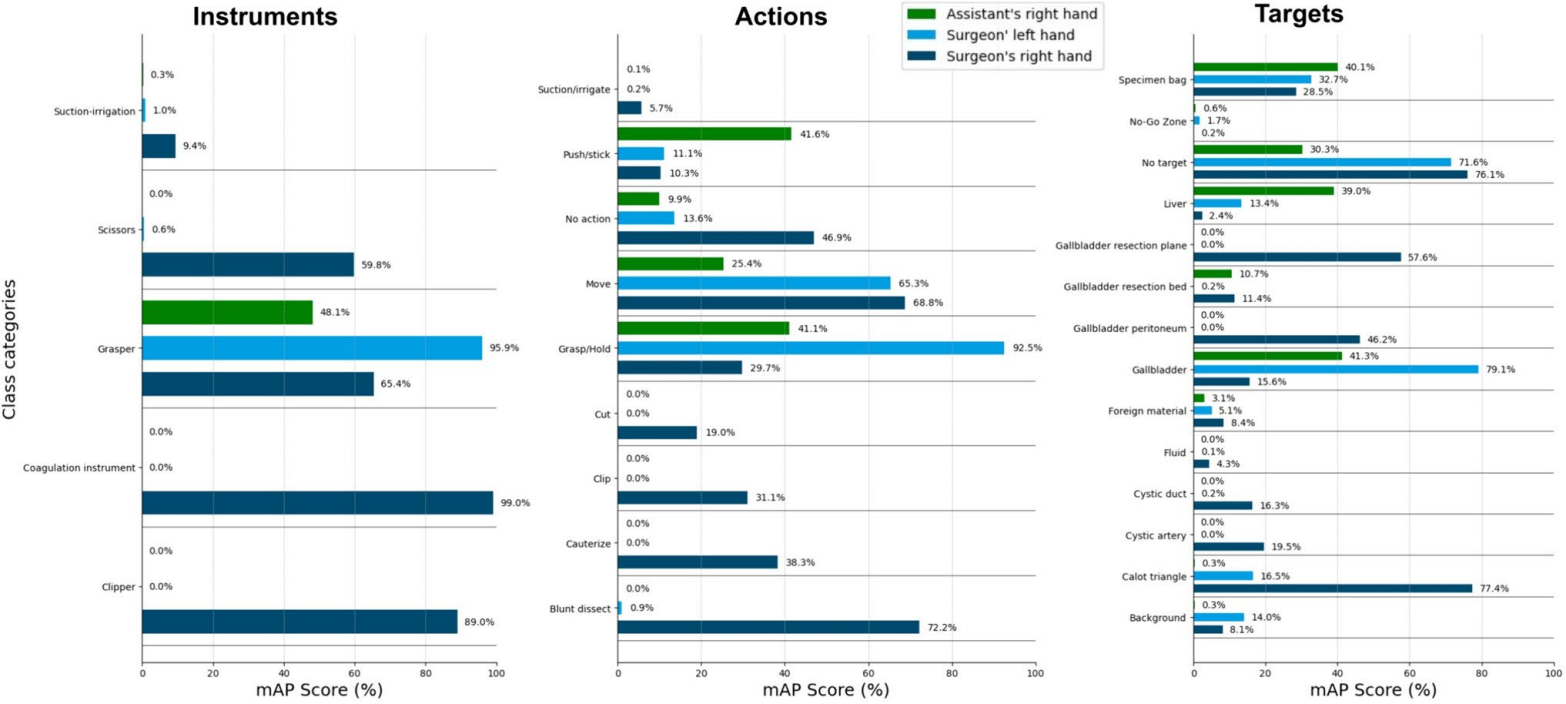
Per-actor class category recognition performance using the HeiCholeActivity dataset for training

In the third experiment with training on our HeiCholeActivity dataset only our own class reduction method and actor separation, the action triplet recognition algorithm scored 49.5% mAP for action triplets of the surgeon’s left hand, 46.8% for action triplets of the surgeon’s right hand and 56.6% for action triplets of the assistant’s right hand. A detailed overview of the per-class performance for each actor can be seen in Fig. 4.

## Discussion

### Activity model for laparoscopic cholecystectomy

Our activity model formalizes the surgical activity workflow aiming at cooperation with collaborative surgical robots and has been applied to annotate real-world videos. Furthermore, our descriptive analysis provides quantitative insights into the surgical workflow, e.g. what the differences between LC on humans and on explanted porcine livers are (Fig. 2) and which activities are performed at which frequency during LC.

### Activity recognition with ML

As of now, activity recognition with machine learning is not clinically applicable yet, probably because of limited data quantity and variability. The algorithm’s cross-validation performance using only the annotated videos on explanted porcine livers as input was higher than using only the annotated videos on humans (Table 2). This could be attributed to the different number of unique action triplet classes in each subset, as more unique classes potentially lead to more class imbalance and lower mAP scores. Passing supplementary separated action, instrument and target labels as well as phase and actor annotations as auxiliary information yielded no particular performance improvement.

In the first experiment, the algorithm’s cross-validation mAP score using all of our own HeiCholeActivity dataset as input (25.7%) was lower than during its participation in the CholecTriplet2022 challenge which used the CholecT45 dataset as input (36.5%) [29]. Possible reasons for this difference in performance can be addressed. While the annotation of all video frames including idle time reflects the actual occurrence of each activity, it introduced a high temporal action triplet class imbalance (Fig. 2). Accentuated by the limited quantity of annotated data, this resulted in a good recognition of frequent classes but poor recognition performance of many infrequent classes. This negatively affected the overall mAP score as the performance for each triplet class has the same weight, but positively affected the top *K* = 5 accuracy as frequent classes were recognized correctly in many frames. Top *K* = 5 accuracy performance should be interpreted cautiously, as it is comparatively high but may not be a suitable metric to measure clinical usability.

Our dataset contains fewer videos than the one used in CholecTriplet2022 (20 vs. 45 videos) and addressing the lower amount of total data and infrequent classes by sampling videos at a higher framerate (3.125 vs. 1 FPS) did not appear to solve this issue. Thus, more videos and video segments should be annotated in the future. The auxiliary use of semantically segmented video frames, which contain rarely targeted but often visible tissues, may also improve performance [42]. Recording videos with multi-modal medical device sensor data, e.g. the electrocautery signal feed, may also improve annotations [51]. Semi-supervised ML approaches may bridge the gap between the availability of surgical video and lack of annotations thereof [52].

In the second experiment, the algorithm cross-validation mAP score using our own HeiCholeActivity dataset as well as CholecT45 as input improved over the score achieved by the algorithm using only CholecT45 as input (37.9% vs. 36.5%) [29, 46]. This improvement is encouraging for further research on state-of-the-art algorithms which achieve 40.6% mAP using only CholecT45 for training [53]. However, the difference in annotation rules between the datasets made the symbolical mapping of our own classes to the classes of CholecT45 partly inconsistent. For example, the target “Calot triangle” was mapped to “cystic_plate” in the CholecT45 dataset. The target “Calot triangle” was often used in our own dataset to annotate dissecting activities in the Calot triangle dissection phase and implicitly includes cystic duct, cystic artery and cystic plate. This is due to the consideration that differentiating the correct target in a causal manner while it was still covered by fatty tissue, and thus potentially false was too ambiguous and subjective for consistent goal-driven annotations. Therefore, “cystic duct” and “cystic artery” can only be annotated when clipped or cut according to our annotation rules. In CholecT45, “cystic duct” and “cystic artery” were also annotated in the Calot triangle dissection phase. This highlights the need for transparent annotation rules across datasets in literature which we address in the Supplementary Material.

In the third experiment, big differences in class performance between actors were noted. For example, the action “Grasp/Hold” was recognized well for the surgeon’s left hand and the target “Calot triangle” was recognized well for the surgeon’s right hand (Fig. 4).

### Limitations

During the development and application of the activity model, several limitations were discovered in regards to the real-time, i.e. causal nature, of the use case of collaborative surgical robotics. A compromise was negotiated between unambiguous start and end events for each activity and a manageable annotation effort during the development and iteration of every annotation rule. For example, the target of a movement can only be roughly estimated until the movement is finished, thus forcing the annotator to change annotations retrogradely if the prior assumed target turns out to be false. This incompatibility between real-time recognition and goal-oriented modeling may be inherent to the ambiguous nature of the prediction of future actions, especially for the use case of collaborative robotic assistance. Modeling in a strictly event-driven manner could cause less causality issues, but would produce either a substantial loss of clinical meaning or a substantially higher annotation effort [39]. Therefore, during model refinement and resulting iterations of the annotation rules, a latency period of one second was introduced to annotate the most probable activity during this timeframe (Supplementary Material, Sect. 2, Action annotation). This allowed causality to be mostly kept, i.e. no significant retrograde correction of the annotated activity to be needed. For the use case of collaborative surgical robots, an input latency of one second is estimated by the authors to be tolerable to reliably understand the surgeon’s workflow early enough in most situations, similarly to a human assistant’s reaction and processing time to see and understand the surgeon’s workflow in real-time. However, to alleviate this latency in the future, muscle activity sensors on the surgeon’s arms may aid to anticipate their next activity [54].

Actors also occasionally performed two actions simultaneously, e.g. the right hand grasper moving to the specimen bag while holding the gallbladder. The activity model captured only the primary action, resulting in the potential loss of clinically relevant information. This could be addressed in future works.

Additionally, “blunt dissect” was found to be an action which included many different dissection techniques with the electric hook, overholt or other instruments. In future works, each dissection technique could become its own class and therefore provide more detailed information on the intraoperative workflow [55]. Similarly, differentiating between the anatomic regions of a targeted tissue could be useful to capture the advancement of the procedure in a more accurate and clinically relevant manner (e.g. dissecting the left, right or center of the Calot triangle and then clipping the proximal or distal end of the cystic duct). This could also be added to the activity model in future works.

### Research steps towards clinical application

LC is a popular benchmark procedure for surgical workflow recognition with ML due to it being a high-volume procedure performed by novice surgeons [21]. However, more complex surgical procedures performed by experienced surgeons such as robot-assisted minimally invasive esophagectomy (RAMIE) are becoming increasingly relevant for surgical workflow recognition due to higher process standardization and clinical need for assistance systems to further improve patient outcomes [56, 57].

Regardless of the selected procedure, high quantity and variation of input data are key for ML algorithms to generalize and be robust for clinical application. Therefore, we add results from a new single-center annotated subset of human LC from a German hospital to the research field. We also created a novel single-center annotated subset of ex vivo porcine LC to facilitate translational research on collaborative surgical robots in pre-clinical development phases. Both subsets share the same surgical activity process model. The model’s classes were mapped to the classes of the CholecT45 dataset in order to use both datasets for algorithm training.

**Fig. 5:**
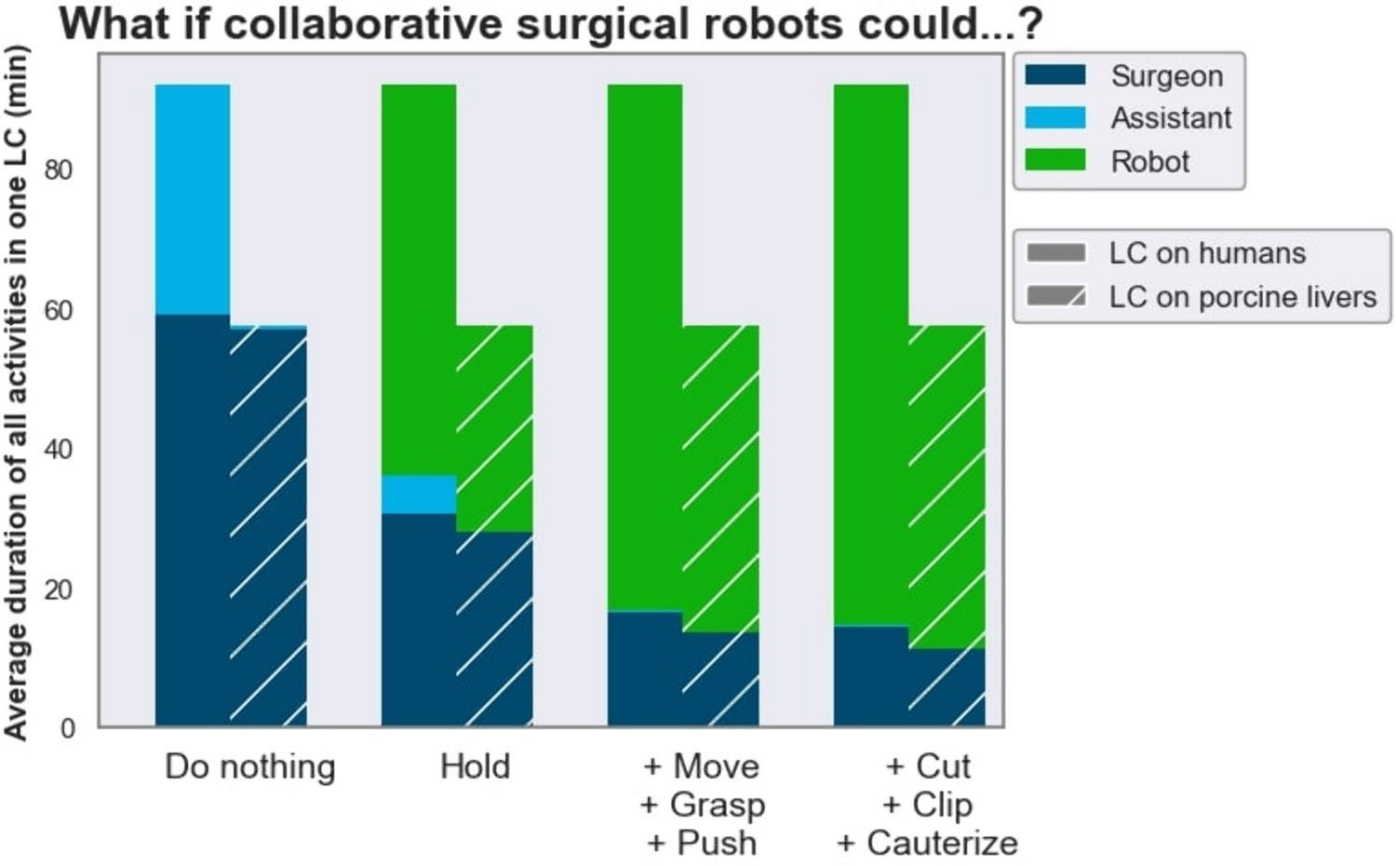
Quantitative impact of collaborative robotics on human–robot-cooperation in laparoscopic cholecystectomy (LC). The plot displays the hypothetical cumulative duration of activities of each stakeholder (surgeon, assistant, robot) across all videos if collaborative surgical robots could autonomously perform increasing sets of specific action tasks. It is assumed that the number of available robots is not limited. Camera holding with the assistant’s left hand was not included, because it was not annotated as an activity in our model and does not involve direct interaction with tissue

If collaborative surgical robots could grasp and hold tissue, 83.5% of the assistant’s tissue interacting tasks (i.e. excluding camera guidance) could be performed by the robot. Thus, collaborative surgical robots may have the potential to lower the workload of surgical staff in LC if they could autonomously perform assisting actions in the future (Fig. 5). If robots took over assisting functions such as using a grasping instrument (moving, pushing, grasping tissue or holding tissue) and controlling the endoscope during LC, they could free up time for the surgical assistant to assist in more complex surgical procedures, work in the ward or rest during on-call night shifts. Conversely, a reliable robotic surgical assistant may lower the hurdle for surgical assistants to start performing basic laparoscopic procedures like LC themselves while the supervising surgeon would not necessarily need to be scrubbed in and assisting.

While robotically automating shorter, more critical actions (such as cutting, clipping, cauterizing) may not save a substantial amount of time in surgery, it may reduce the surgeon’s daily mental stress if performed reliably [58].

The requirements for state-of-the-art data input for ML compelled us to preprocess the dataset. This resulted in decreased data complexity and introduced feature selection as a type of data leak [59]. However, the authors believe that the data’s complexity reflects the activity model’s clinical relevance. In regards to enabling collaborative surgical robotics, the data’s clinical relevance is crucial, and thus should not be simplified but preserved. The authors consider that such collaborative surgical robots will require comparable workflow recognition capabilities to that of surgeons for seamless and complex real-time assistance, which does not leave space for trade-offs in data complexity and may require further improvements in performance of ML algorithms.

Concerning the practical use of the activity model, we estimate the actual annotating time for all 20 videos to have taken a total of over 500 working hours, including the learning curve and iterations, despite the specified video selection criteria. Therefore, we consider annotation effort to be a bottleneck for the further use of machine learning algorithms for activity recognition. The resulting selection bias may have resulted in the collection of less diverse data and less edge cases. Those bottlenecks could be addressed by the use of foundation models during the annotation process [60]. However, foundation models designed for workflow recognition are lacking. Label interpolation could make use of annotated time segments in order to augment the quantity of annotated data. Perceived annotation effort could also be reduced if a modern, free and gamified video annotation software were developed [61]. Less annotation effort would reduce selection bias and potentially enable multi-center randomized video collection for surgical action annotation. This would also enable the annotating capacity to be scaled up to improve intra- and inter-rater reliability of the annotations.

Finally, future works could contain more surgical settings, e.g. robot-assisted LC on humans and LC on living pigs. This could improve the generalization of ML-based surgical action recognition algorithms during the preclinical development of collaborative surgical robots.

Importantly, considerations on accountability and liability in the use of collaborative surgical robots leveraging automated processes need to be taken into account already during the development process. A level of autonomy (LoA) needs to be clearly defined in each development stage and comply with national and international medical device regulations before entering the market. However LoA definitions lack consistency in literature and need a unified approach [11, 62, 63]. Especially, a potential of harm to patients needs to be avoided at all costs. Determining the liability of the main stakeholders, mainly between the surgical staff and the robot manufacturer, when complications for the patient arise, can pose a challenge when the system is tightly integrated into the surgical workflow and responsibilities are not clearly defined [64].

## Conclusion

This paper introduces an activity model of laparoscopic cholecystectomy in different surgical settings. Its first application with video annotation and its use for machine learning-based action triplet recognition are presented. The activity model accurately captures clinically relevant information on the fine granular surgical workflow and shows the potential of collaborative surgical robots in the operating rooms of the future. However, the gap between the constraints of machine learning approaches and the requirements of surgeons for the use case of collaborative surgical robotics needs to be bridged in future works.

### Supplementary Information

Below is the link to the electronic supplementary material.

Supplementary file1 (DOCX 58 KB)

## Data Availability

The raw video data and annotations that support the findings of this study are available from the corresponding author upon reasonable request.
