# Supplementary material for: A surgical activity model of laparoscopic cholecystectomy for co-operation with collaborative robots
Source: Surg Endosc. 2024 Jun 13;38(8):4316–28. doi: 10.1007/s00464-024-10958-w (PMC11289174; doi:10.1007/s00464-024-10958-w)
Supplement: Supplementary file 1 — Supplementary file1 (DOCX 58 KB) [file 464_2024_10958_MOESM1_ESM.docx]

Supplementary Material

# Section 1: Class selection

## Phases

| Class |
| --- |
| Preparation* |
| Calot triangle dissection* |
| Clipping and cutting of the vessels* |
| Gallbladder dissection* |
| Gallbladder packaging* |
| Cleaning and coagulation* |
| Gallbladder removal |

## Instrument presence

| Class |
| --- |
| Invisible |
| Visible |

## Actors

| Stakeholder | Class |
| --- | --- |
| Operating surgeon | Right-hand of surgeon |
|  | Left-hand of surgeon |
| Surgical assistant | Right-hand of assistant |

The left-hand of assistant is only used for camera guidance during LC and is therefore not listed.

## Instruments

| Class category | Class | Remarks for annotation |
| --- | --- | --- |
| Grasper* | Atraumatic grasper* |  |
|  | Atraumatic grasper short* |  |
|  | Overholt* |  |
|  | Crocodile forceps* |  |
|  | Swab grasper* | Holding the swab is inherent to this instrument and not counted as the primary action. |
|  | Pointed forceps* |  |
|  | Curved atraumatic grasper* |  |
|  | Toothed grasper* |  |
|  | Fenestrated toothed grasper* |  |
|  | Tiger mouth forceps* |  |
|  | Claw forceps* |  |
|  | Flat grasper* |  |
| Clipper* | Clip-applier (metal)* | Sweeping along the cystic artery or cystic duct with the clip jaws counts as ”Move”. |
|  | Clip-applier (Hem-O-Lok)* |  |
| Coagulation instrument* | Electric hook* |  |
|  | LigaSure* |  |
|  | Argon beamer* |  |
|  | Instrument with electric cable |  |
| Scissors* | Scissors* |  |
| Suction-  irrigation* | Suction-irrigation* |  |
| Non-relevant instrument | Stapler* |  |
|  | Finger | Annotated when an actor pushes their finger inside the abdominal cavity through the incisions and when a drainage is inserted. |
|  | Specimen bag cartridge |  |
|  | Undefined instrument shaft* |  |

## Actions

| Class category | Class | Clinical priority (see Section 3) |
| --- | --- | --- |
| No action | No action | High |
| Grasp/Hold | Grasp* | Moderate |
|  | Hold* |  |
| Cut | Cut* | High |
| Clip | Clip* | High |
| Blunt dissect | Blunt dissect | High |
| Cauterize | Cauterize | High |
| Suction/irrigate | Irrigate | Moderate |
|  | Suction |  |
| Push/stick | Stick | Low |
|  | Push |  |
| Move | Move | High |

## Targets

| Class category | Class | Remarks for annotation |
| --- | --- | --- |
| No target | No target |  |
| Gallbladder | Gallbladder** |  |
| Gallbladder peritoneum | Gallbladder peritoneum | Includes only the visceral peritoneum of the gallbladder. |
| Gallbladder resection plane | Gallbladder resection plane | Includes only fibrous tissue attaching gallbladder to liver wrapped under gallbladder peritoneum. |
| Gallbladder resection bed | Gallbladder resection bed** |  |
| Calot triangle | Calot triangle | ”Triangle formed by the cystic duct, the common hepatic duct, and inferior edge of the liver” [1].  Also includes fibrous tissue wrapped under gallbladder peritoneum located at the inferior edge of the liver during Calot triangle dissection.  Also includes the cystic duct and cystic artery if the annotation criteria below do not apply. |
| Liver | Liver** |  |
| Cystic duct | Cystic duct** | Annotated when clipped, cut (with cauterization or not), injured or pressed by overholt. |
| Cystic artery | Cystic artery** | Annotated when clipped, cut (with cauterization or not) or injured. |
|  | Unnamed vessel* | Annotated if more than one artery is found and needs to be clipped [2]. |
| Background | Fatty tissue** | Includes adhesions. |
|  | Round and falciform ligament** |  |
|  | Abdominal wall and diaphragm** |  |
|  | Undefined target |  |
| Fluid | Blood pool** |  |
|  | Bile |  |
|  | Irrigation fluid |  |
| Specimen bag | Specimen bag** |  |
| Foreign material | Instrument as target | When an instrument targets another. |
|  | Trocar** |  |
|  | Drainage** | The instrument moving the drainage is the finger inserting the drainage from outside the abdomen. |
|  | Gauze** |  |
|  | Clip unit** | Does not include the cystic artery, cystic duct or Calot triangle as opposed to HeiSurF definition. |
| No-Go Zone | Gastrointestinal tract** |  |
|  | Hilum of liver** | Includes the portal triad and fatty tissue wrapping it. |

**As defined in the HeiChole benchmark challenge* [2]*.*

*** As defined in the HeiSurF benchmark challenge* [3]*.*

# Section 2: Annotation rules

## Definitions

The definitions are based on the newest SAGES recommendations [4] with the exception of the definition of a phase. Here, we use the term “phase” for what was defined by SAGES as “step”.

### Phase, Task

The intraoperative phases consist of tasks. The granularity level “Task” is not annotated. The table below shows the authors’ interpretation of phases and tasks for laparoscopic cholecystectomy, based on previous work [2, 5]:

| **Phase ID** | **Phase** | **Task ID** | **Task** |
| --- | --- | --- | --- |
| P0 | Preparation | T0.1 | Insert 10 mm port (umbilical) for left-hand of assistant endoscope |
|  |  | T0.2 | Perform diagnostic laparoscopy of abdomen |
|  |  | T0.3 | Insert 10 mm port (left upper quadrant) for right-hand of surgeon |
|  |  | T0.4 | Insert 5 mm port (right lateral) for left-hand of surgeon |
|  |  | T0.5 | Insert 5 mm port (epigastric) for right-hand of assistant |
| P1 | Calot triangle dissection | T1.1 | Dissect adhesions to gallbladder (optional depending on patient) |
|  |  | T1.2 | Dissect & mobilize Hartmann’s pouch |
|  |  | T1.3 | Dissect & isolate cystic duct |
|  |  | T1.4 | Dissect & mobilize cystic artery |
|  |  | T1.5 | Perform critical view of safety (CVS) |
| P2 | Clipping and cutting of the vessels | T2.1 | Place clip at gallbladder end of cystic duct |
|  |  | T2.2 | Place 2 clips on proximal end of cystic duct proximal to incision on cystic duct |
|  |  | T2.3 | Place 2 clips on proximal end of cystic artery |
|  |  | T2.4 | Place clip on distal end of cystic artery |
|  |  | T2.5 | Cut cystic artery |
|  |  | T2.6 | Cut cystic duct |
| P3 | Gallbladder dissection | T3.1 | Dissect medial side of gallbladder up to fundus |
|  |  | T3.2 | Dissect lateral side of gallbladder up to fundus |
|  |  | T3.3 | Separate undersurface of gallbladder from liver |
|  |  | T3.4 | Secure any bleeding from liver bed |
| P4 | Gallbladder packaging | T4.1 | Insert retrieval bag |
|  |  | T4.2 | Place gallbladder inside bag |
| P5 | Cleaning and coagulation | T5.1 | Check & coagulate any bleeding areas |
|  |  | T5.2 | Check cystic artery stump & clips |
|  |  | T5.3 | Check cystic duct & clips |
|  |  | T5.4 | Irrigate & suction operative field |
|  |  | T5.5 | Place drain under liver bed (optional depending on patient and critical events) |
| P6 | Gallbladder retraction | T6.1 | Extract bag containing gallbladder |
| P7 | Ending of the operation | T7.1 | Remove epigastric and lateral ports |
|  |  | T7.2 | Check port sites |
|  |  | T7.3 | Release CO2 from abdomen |
|  |  | T7.4 | Remove umbilical port |
|  |  | T7.5 | Suture port sites |
|  |  | T7.6 | Clean port-site areas |
|  |  | T7.7 | Place dressings over port sites |

### Activity

An activity is a well-defined surgical motion unit [6]. While this granularity level is not explicitly included in the SAGES consensus recommendations, we define the activity as being on the same level as the action, but with contextual information [7, 8]. Concretely, it is a tuple consisting of four items. The behavior between the four items can be put as follows:

„The actor performs an action on the target with an instrument at a given timeframe.“

Example: “The surgeon's left-hand grasps the gallbladder with an atraumatic grasper at 3:26-3:28.”

**Actor:** This specifies who performs the action (organizational).

**Instrument:** This specifies the tool in use (operational). It is manipulated by the actor.

**Instrument presence:** This indirect contextual information specifies whether the instrument is visible in the video or not (informational).

**Action:** This specifies what is being done and consists of gestures (functional). It is represented by a verb.

**Target:** This specifies what tissue is being manipulated (spatial).

**Timeframe:** This specifies when the action takes place (behavioral).

### Gesture

A gesture is one trajectory with no semantics [6].

This granularity level is not being annotated.

## Activity annotation rules

### General annotation rules

1. These annotation rules have been designed for activity annotation of laparoscopic cholecystectomy on humans and on explanted porcine livers.
2. An activity always consists of: one actor, one action, one instrument, one target and a given timeframe, the instrument being present or not. Thus, every activity is unique.
3. Only intraoperative activities that take place in the abdomen or laparoscopic box can be annotated.
4. Only hierarchical tasks, i.e. activities that are interpretable from surgical video, shall be annotated.
5. If an actor performs two activities at the same time (e.g. pushing the liver while dissecting the gallbladder peritoneum), the primary activity shall be annotated, i.e. the one that is more relevant to the accomplishment of the surgical objective (in this example dissecting the gallbladder peritoneum). This is left to the annotator’s expertise.

### Time-frame annotation

1. A frame represents a unique timestamp of the procedure.
2. The annotation time starts together with video start (0:00:00) and ends with video end.
3. One second represents 25 frames in the ANVIL annotation software.
4. An activity starts on the first frame it takes place and stops on the frame directly after the last frame it takes place in the ANVIL annotation software.
5. There can be several activities happening in one frame, but only one activity per frame per actor is annotated.
6. Two activities which are directly consecutive but do not differ other than by the time-frame shall become one (fusion rule).

### Actor annotation

1. An actor corresponds to one hand of the operating stakeholders, the stakeholders being the surgeon and surgical assistant in laparoscopic cholecystectomy.
2. The annotator shall estimate which hand is performing an activity and annotate accordingly, no matter through which trocar the hand is operating.

### Instrument annotation

1. Picture examples for each instrument can be found in the HeiChole Benchmark study [2]
2. As specified in the HeiChole Benchmark study: “if the instrument shaft enters the abdomen without more than 50% of its tip having been visible before, it is referred to as the undefined instrument shaft [...]. Three exceptions are the suction-irrigation, stapler and the clippers, as these instruments have characteristic shafts.” [2].
3. If the instrument stays in the abdomen and only the shaft is seen, it is not annotated as an undefined instrument shaft but as the specific instrument until it is pulled out of the abdomen again.

### Instrument presence annotation

1. Instruments are either visible or not when activities are performed with them.
2. Visibility of each actor’s instruments shall be annotated accurately on a per-frame basis.
3. As specified in the HeiChole Benchmark study: “an instrument is considered visible as soon as its characteristic instrument tip appears in the image. The annotation continues when the tip disappears later and only the shaft of the instrument remains visible. An example is the disappearance of the instrument tip of the electric hook behind tissue during dissection. Most importantly, the shaft should be clearly associated with an instrument tip.” [2].
4. An activity is invisible when the instrument cannot be seen in the frame (fat droplets and other objects are ignored and only the metal tip and shaft count). This is the case mostly when:
   1. It is out of image or so near the edge of the image that it is indistinguishable from the rest of the scene for a human
   2. It is covered by something (tissue or instrument) which makes it indistinguishable from the rest of the scene
   3. It lies in the shadow of the laparoscopic light or in the background so as to not be distinguishable from the rest of the scene for a human anymore (for example no light reflection of the metal tip and no silhouette visible)
   4. Any other special case emerges that makes the instrument indistinguishable from the rest of the scene for a human (e.g. smoke)
5. Instruments that are only visible inside trocars count as invisible.
6. Shadows, glows, and mirroring of instruments on shiny tissue do not qualify them as being visible.

### Target annotation

1. Every annotated activity should include a target, except activities that consist of the actions “Move”, “No action” and “Grasp” when grasping fails.
2. If there is an active bleeding being targeted, it is annotated with the location of the bleeding and not as an “unnamed vessel” (except if it is an unnamed vessel).

### Action annotation

1. Every annotated action belongs either to the stoptimer category or to the one second category.
2. Stoptimer category: activities with the actions “Grasp”, “Hold”, “Cut”, “Clip”, “Cauterize”, “Irrigate”, “Suction” and “Stick”. The aforementioned activities shall be annotated as accurately as possible on a per-frame basis.
3. One second category: activities with the actions “Blunt dissect”, “Push”, “Move”, “No action”. For the aforementioned activities, following rules apply:
   1. If an activity A is followed by an activity B with the action “Move” or “No action” for less than a second (< 1s) before the activity A is repeated, it can directly be continuously annotated (repetition rule).
   2. If an activity has “No target” for less than a second (< 1s) before it has the same action with a target (again), it shall be continuously annotated with this target (lag rule).
   3. If an activity has “No action” for less than a second (< 1s) before it has “Move”, it shall be continuously annotated with ”Move” (pause rule).
   4. If the instrument becomes invisible during activity A for less than a second (< 1s) and the annotator cannot estimate what happens during that time, activity A is annotated until it is visible again or until activity B can be estimated during invisibility (invisibility rule). If the action is “Move” or “No action” and the invisibility lasts for more than a second, nothing shall be annotated for that time period and the visibility rule from the HeiChole Benchmark study (see subsection “Instrument presence”) is reset because a new instrument could be introduced.

## Specific action annotation rules

|  | Grasp |
| --- | --- |
| Start | When the instrument jaws begin to be fluidly closed (even if it is further away, but this is left to the annotator's estimation). |
| End | When the instrument jaws stop closing because of the thickness of the tissue or when the instrument jaws are fully closed. |
| Notes | If only air is being grasped (from the beginning or just in the end), then “No target” shall be annotated.  If the instrument does not fully close when grasping with no target (making it a “fake” grasp), the action is not counted as grasping. |
| Category | Stoptimer |

|  | Hold |
| --- | --- |
| Start | If the target was grasped before: directly after the “Grasp” action is successfully completed.  If the target is already being held when the activity starts (e.g. when a forceps already holding a gauze enters the surgical field): in the first frame it is seen. |
| End | As soon as the grasper begins to release its grip or when tissue starts slipping out of the grip.  If an instrument is not seen releasing a target and nothing indicates it released it, it is assumed that it continues holding. Exception: if a target is being pulled out of the abdomen while performing the holding action, the activity stops after the last frame the target and instrument are visible [2].  If an instrument is not seen releasing a target but the target moves in a way that indicates the instrument does, the activity stops. |
| Notes | If the target sticks to the instrument after release, it should be annotated as the action “Stick”. |
| Category | Stoptimer |

|  | Cut |
| --- | --- |
| Start | When the instrument starts to fluidly close around a target. |
| End | When the instrument is fully closed. |
| Notes | Cutting air is annotated as “Cut” with “No target”.  Dissecting with open scissors is not cutting. It shall be annotated as “Blunt dissect”. |
| Category | Stoptimer |

|  | Clip |
| --- | --- |
| Start | When the instrument starts to close around a target. |
| End | When the clipper begins to release its grip after the application of a clip. |
| Notes |  |
| Category | Stoptimer |

|  | Blunt dissect |
| --- | --- |
| Start | When the instrument tip touches the target with the intent to dissect it. |
| End | When the instrument doesn’t touch the target anymore. |
| Notes | Includes the characteristic movements described by Ma et al. [9]. |
| Category | One second |

|  | Cauterize |
| --- | --- |
| Start | When the instrument is visually estimated to start cauterizing the target. |
| End | When the instrument is visually estimated to stop cauterizing the target. |
| Notes |  |
| Category | Stoptimer |

|  | Irrigate |
| --- | --- |
| Start | When water is first seen applied by the actor or the water level is rising with the irrigation below the water surface. |
| End | When no water is actively coming out of the instrument anymore. |
| Notes | Water dripping out of the instrument after irrigation does not count as irrigating. |
| Category | Stoptimer |

|  | Suction |
| --- | --- |
| Start | When liquid or tissue starts disappearing because of the instrument or when the instrument moves in a pouch where liquid is present frequently in a way that makes suctioning apparent (annotator estimation). |
| End | When the instrument is not touching a liquid anymore or no tissue or liquid is disappearing anymore because of the instrument. |
| Notes | “Suction” is vacuuming liquid into the suction-irrigation instrument. |
| Category | Stoptimer |

|  | Stick |
| --- | --- |
| Start | When a target still adheres to the instrument even though the preceding action ended. |
| End | When another action can be annotated, namely when the target does not stick anymore or the actor starts performing another action. |
| Notes | When a target sticks after or during blunt dissection, it still counts as blunt dissection and not as sticking. |
| Category | Stoptimer |

|  | Push |
| --- | --- |
| Start | When the instrument starts touching a target to hold it in some way (in the case of a grasping instrument: when it hasn’t closed its jaws around the target). |
| End | When the instrument stops pushing the target (not last contact). |
| Notes |  |
| Category | One second |

|  | Move |
| --- | --- |
| Start | when the instrument starts moving or rotating but none of the above actions can be defined. |
| End | when another action can be annotated or when the instrument disappears for more than one second. |
| Notes | An instrument is considered as moving when the instrument changes position significantly. Unintentional hand movements by the operating hand while moving are included. Little movements and trembling that do not lead to a significant change of instrument position do not have to count as moving and can count as “No action”.  Palpating tissue can be included in the action “Move” when it does not lead up to the action “Blunt dissect” (e.g. touching tissue lightly without dissecting in order to cauterize). |
| Category | One second |

|  | No action |
| --- | --- |
| Start | When no other action can be attributed to the actor but an instrument can be seen. |
| End | When another action starts or the instrument isn’t seen anymore. |
| Notes | “No action” is when none of the actions above are happening. Mostly, this is the case when an instrument is not even moving or not moving significantly. By definition, “No action” is annotated with “No target”. |
| Category | One second |

# Section 3: Class reduction

After the annotation process, the number of occurring triplet combinations needed to be reduced, as there were too many different combinations for the machine learning algorithm used. Thus, classes were either removed (e.g. stapler) or grouped (e.g. suction and irrigate to suction/irrigate) by clinical similarity, relevance and practices in previous literature [2, 10, 11]. For each decision, an explanation is given in the table below.

| **Reduction process** | **Reduced classes** | **Clinical reason for reduction** | **Data-based reason for reduction** |
| --- | --- | --- | --- |
| Categorized then removed:  non-relevant instruments | *Stapler* | Irrelevant for regular procedures as this instrument is only used in rare and difficult cases of LC. | No occurrence in the annotated data. |
|  | *Finger* | Irrelevant to the completion of the intraoperative surgical phases as the finger is only seen occasionally after the final gallbladder retraction to prepare for abdominal suturing. | Rare occurrence in the annotated data. |
|  | *Specimen bag cartridge* | The specimen bag was mostly inserted without being shown in video because it is soft material and may not cause adverse events. Therefore, it was considered to not be clinically relevant. | Rare and very short occurrence in the annotated data. |
|  | *Undefined instrument shaft* | Edge case during instrument insertion that was considered to be of low clinical importance for surgical activity recognition. | Rare and very short occurrence in the annotated data. |
| Categorized:  actions | *Grasp* + *Hold*  *→*  *Grasp/Hold* | Distinction considered as being of moderate clinical priority for surgical action recognition. Both actions are often consecutive by definition and therefore could be grouped. | Frequent but very short occurrence of *Grasp* in the annotated data. |
|  | *Suction* + *irrigate*  *→*  *Suction/irrigate* | Considered as being of low clinical priority for surgical action recognition. Both actions are often used in alternation and therefore could be grouped. | Rare occurrence in the annotated data. |
|  | *Stick* + *Push*  *→*  *Stick/Push* | Considered as being of low clinical priority for surgical action recognition. Both actions mean holding the target without grasping by definition and therefore could be grouped. | Rare and very short occurrences in the annotated data. |
| Categorized:  targets | *Unnamed vessel + cystic artery*  *→*  *Cystic artery* | An unnamed vessel is an accessory artery supplying the gallbladder. It is therefore also an artery by definition and could be grouped. | Rare occurrence in the annotated data. |
|  | *Fatty tissue* + *round and falciform ligament* + *abdominal wall and diaphragm* + *undefined target*  →  *Background* | All classes represent tissue which often is irrelevant to the completion of the intraoperative surgical phases and rarely lead to adverse events if targeted. Therefore, they were grouped. | Rare occurrence in the annotated data. |
|  | *Blood pool* + *bile* + *irrigation fluid*  →  *Fluid* | All classes are fluids by definition and may only occur during *suction-irrigate*, which is of moderate clinical priority. | Rare occurrence in the annotated data. |
|  | *Instrument as target* + *trocar* + *drainage* + *gauze* + *clip unit*  *→*  *Foreign material* | Targeting these materials may rarely lead to adverse events. Additionally, these are foreign materials introduced in the patient body by definition and therefore could be grouped. | Rare occurrence in the annotated data. |
|  | *Gastrointestinal tract + Hilum of liver*  *→*  *No-Go-Zone* | Tissue which is often irrelevant to the completion of the intraoperative surgical phases but could lead to adverse events if targeted. Therefore, they could be grouped. | Rare occurrence in the annotated data. |

# References

1. The SAGES Safe Cholecystectomy Program - Strategies for Minimizing Bile Duct Injuries. In: SAGES. https://www.sages.org/safe-cholecystectomy-program/. Accessed 2 Sep 2023

2. Wagner M, Müller-Stich B-P, Kisilenko A, Tran D, Heger P, Mündermann L, Lubotsky DM, Müller B, Davitashvili T, Capek M, Reinke A, Reid C, Yu T, Vardazaryan A, Nwoye CI, Padoy N, Liu X, Lee E-J, Disch C, Meine H, Xia T, Jia F, Kondo S, Reiter W, Jin Y, Long Y, Jiang M, Dou Q, Heng PA, Twick I, Kirtac K, Hosgor E, Bolmgren JL, Stenzel M, von Siemens B, Zhao L, Ge Z, Sun H, Xie D, Guo M, Liu D, Kenngott HG, Nickel F, Frankenberg M von, Mathis-Ullrich F, Kopp-Schneider A, Maier-Hein L, Speidel S, Bodenstedt S (2023) Comparative validation of machine learning algorithms for surgical workflow and skill analysis with the HeiChole benchmark. Med Image Anal 86:102770. https://doi.org/10.1016/j.media.2023.102770

3. Wagner M, Bodenstedt S (2021) Endoscopic Vision Challenge (EndoVis) 2021: HeiChole Surgical Workflow Analysis and Full Scene Segmentation (HeiSurF). https://www.synapse.org/heisurf. Accessed 27 Oct 2021

4. Meireles OR, Rosman G, Altieri MS, Carin L, Hager G, Madani A, Padoy N, Pugh CM, Sylla P, Ward TM, Hashimoto DA, the SAGES Video Annotation for AI Working Groups (2021) SAGES consensus recommendations on an annotation framework for surgical video. Surg Endosc 35:4918–4929. https://doi.org/10.1007/s00464-021-08578-9

5. Sarker SK, Hutchinson R, Chang A, Vincent C, Darzi AW (2006) Self-appraisal hierarchical task analysis of laparoscopic surgery performed by expert surgeons. Surg Endosc 20:636–640. https://doi.org/10.1007/s00464-005-0312-5

6. Lalys F, Jannin P (2014) Surgical process modelling: a review. Int J Comput Assist Radiol Surg 9:495–511. https://doi.org/10.1007/s11548-013-0940-5

7. Jablonski S, Bussler C (1996) Workflow Management: Modeling Concepts, Architecture, and Implementation

8. Neumuth T, Jannin P, Strauss G, Meixensberger J, Burgert O (2009) Validation of Knowledge Acquisition for Surgical Process Models. J Am Med Inform Assoc 16:72–80. https://doi.org/10.1197/jamia.M2748

9. Ma R, Ramaswamy A, Xu J, Trinh L, Kiyasseh D, Chu TN, Wong EY, Lee RS, Rodriguez I, DeMeo G, Desai A, Otiato MX, Roberts SI, Nguyen JH, Laca J, Liu Y, Urbanova K, Wagner C, Anandkumar A, Hu JC, Hung AJ (2022) Surgical gestures as a method to quantify surgical performance and predict patient outcomes. Npj Digit Med 5:187. https://doi.org/10.1038/s41746-022-00738-y

10. Kotsiantis SB, Zaharakis ID, Pintelas PE (2006) Machine learning: a review of classification and combining techniques. Artif Intell Rev 26:159–190. https://doi.org/10.1007/s10462-007-9052-3

11. Nwoye CI, Gonzalez C, Yu T, Mascagni P, Mutter D, Marescaux J, Padoy N (2020) Recognition of Instrument-Tissue Interactions in Endoscopic Videos via Action Triplets. pp 364–374
